# Retraction: Long Noncoding RNA RGMB-AS1 Indicates a Poor Prognosis and Modulates Cell Proliferation, Migration and Invasion in Lung Adenocarcinoma

**DOI:** 10.1371/journal.pone.0257517

**Published:** 2021-09-14

**Authors:** The PLOS ONE Editors

Following the publication of this article [1], concerns were raised regarding results presented in Figs 4 and 5, including similarity between results presented in these figures and figures presented previous and subsequent publications. Specifically,

- The A549 NC panel of Fig 4C [1] appears similar to the KYSE30 Blank panel of [2] when flipped horizontally.
- The following mice appear similar, despite being used to represent experiments using different cell lines and/or different experimental conditions:
  ○ Fig 5A A549 Blank [1] and Fig 2G EC9706 Blank [3] when flipped horizontally.
  ○ Fig 5A A549 NC [1] and Fig 2G EC9706 NC [3] when flipped horizontally.
  ○ Fig 5A A549 si-IncRNA [1] and Fig 4A 5-FU G648C [4].
  ○ Fig5B SPC-A-1 Blank [1] and Fig4B A549 Blank [5] when flipped horizontally.
  ○ Fig 5B SPC-A-1 Si-IncRNA [1] and Fig 5A A549 miR-203 [6].
- The SPC-A-1 cell line used in this study is a contaminated cell line; originally thought to be a lung adenocarcinoma cell line, but since shown to be a HeLa derivative [7].

The corresponding author stated that the observed similarities are the result of mismanagement of archived pictures of xenograft experiments but stands by the reliability and veracity of the original results. The corresponding author indicated that the original data underlying the published results are no longer available, and provided replacement images for Figs 4C, 5A, and 5B. However, in the absence of the original data underlying the results presented in this study, the replacement figures are not sufficient to resolve the abovementioned concerns.

In light of the above issues pertaining to the reliability of the reported results, the *PLOS ONE* Editors retract this article.

GZhao agreed with the retraction and apologised for the issues with the published article. PL, GZhang, JL, RY, SC, SW, FZ, YB, HZ, YW, SD, XC, and QS either did not respond directly or could not be reached.

## References

[1] Li P, Zhang G, Li J, Yang R, Chen S, Wu S, et al. (2016) Long Noncoding RNA RGMB-AS1 Indicates a Poor Prognosis and Modulates Cell Proliferation, Migration and Invasion in Lung Adenocarcinoma. PLoS ONE 11(3): e0150790. 10.1371/journal.pone.0150790 26950071 PMC4780832

[2] He W, Feng J, Zhang Y, Wang Y, Zang W, and Zhao G. (2016) microRNA-186 inhibits cell proliferation and induces apoptosis in human esophageal squamous cell carcinoma by targeting SKP2. Lab Invest 96, 317–324. 10.1038/labinvest.2015.134 26568291

[3] Zang W, Wang T, Wang Y, Chen X, Du Y, Sun Q, et al. (2016) Knockdown of long non-coding RNA TP73-AS1 inhibits cell proliferation and induces apoptosis in esophageal squamous cell carcinoma. Oncotarget. 7: 19960–19974. 10.18632/oncotarget.6963 26799587 PMC4991431

[4] Wang Y, Sun Q, Guo W, Chen X, Du Y, Zang W, et al. (2016) G648C variant of DNA polymerase β sensitizes esophageal cancer to chemotherapy. Tumor Biol. 37, 1941–1947. 10.1007/s13277-015-3978-x 26334617

[5] Bai Y, Lu C, Zhang G, Hou Y, Guo Y, Zhou H, et al. (2017) Overexpression of miR-519d in lung adenocarcinoma inhibits cell proliferation and invasion via the association of eIF4H. Tumor Biology. 10.1177/1010428317694566 28351305

[6] Cheng R, Lu C, Zhang G, Zhang G, and Zhao G. (2017) Overexpression of miR-203 increases the sensitivity of NSCLC A549/H460 cell lines to cisplatin by targeting Dickkopf-1. Oncology Reports, 37, 2129–2136. 10.3892/or.2017.5505 28350100

[7] Ye F, Chen C, Qin J, Liu J, and Zheng C. (2015) Genetic profiling reveals an alarming rate of cross-contamination among human cell lines used in China. FASEB J. 29(10):4268–72. doi: 10.1096/fj.14-266718. . 26116706

